# Correlation of Serum Albumin Levels With the Severity of Sepsis Among Intensive Care Unit Patients

**DOI:** 10.7759/cureus.71411

**Published:** 2024-10-14

**Authors:** Muhammad Ammar Ali, Muhammad Tahir Raza, Saqib Majeed, Urooj Tahir, Waseem Ahmad, Mohid Bin Tahir, Rana Shahzaib Ali, Aleeza Afzal, Muhammad Qasim Hasan, Muhammad Hassan, Sana Liaquat, Tayyab Mumtaz Khan

**Affiliations:** 1 Internal Medicine, Rawalpindi Medical University, Rawalpindi, PAK; 2 Emergency, University Hospitals Coventry and Warwickshire, Coventry, GBR; 3 Colorectal Surgery, The Royal London Hospital, London, GBR; 4 Internal Medicine, Jinnah Hospital, Lahore, PAK; 5 Emergency Medicine, Sheikh Zayed Medical College and Hospital, Rahim Yar Khan, PAK; 6 Internal Medicine, Allama Iqbal Medical College, Lahore, PAK; 7 Internal Medicine, Sheikh Zayed Medical College and Hospital, Rahim Yar Khan, PAK; 8 Orthopaedic Surgery, Rawalpindi Medical University, Rawalpindi, PAK

**Keywords:** albumin, care, correlation, intensive, patients, sepsis, serum, severity, unit

## Abstract

Background

Sepsis is a critical and potentially fatal medical condition characterized by significant illness and death rates. Early recognition and assessment of sepsis severity are vital for its optimal management. Determination of its severity by Sequential Organ Failure Assessment (SOFA) and Acute Physiology and Chronic Health Evaluation (APACHE) II, is quite a complex process as these score systems require complex and costly investigations. Therefore, this study was designed to determine the predictive capacity of serum albumin levels for the severity of sepsis in intensive care unit (ICU) patients.

Methods

This cross-sectional study was carried out on 201 ICU-admitted patients with diagnosed sepsis at Benazir Bhutto Hospital (BBH), Rawalpindi, Pakistan from March 2022 to April 2023. Recruitment of patients was performed through consecutive sampling and predefined inclusion and exclusion criteria. Prior to the data collection, ethical approval and informed consent were obtained. Data was gathered via a self-designed proforma. SOFA score was applied for the determination of the severity of sepsis. Patients were categorized into three groups based on sepsis severity (SOFA score). Data analysis was done in the Statistical Package for the Social Sciences (SPSS) version 25. Descriptive and inferential statistics compared study variables. Pearson's correlation and a simple linear regression model were used to assess the relationship between serum albumin levels and sepsis severity and the predictive capacity of serum albumin levels for sepsis severity respectively. The statistical significance of the p-value was set at less than 0.05.

Results

Among the 201 patients, 64 (31.84%) had sepsis, 98 (48.75%) had severe sepsis, and 39 (19.41%) had septic shock. Hypoalbuminemia was present among 119 (59.20%) patients while 82 (40.80%) patients had normal albumin levels. Significant differences were found in the total bilirubin, serum creatinine, platelet count, PaO2/FiO2 ratio, mean arterial pressure, Glasgow Coma Scale score, SOFA score, serum albumin level, and the prevalence of normal and low albumin levels across three study groups (p < 0.05). Pearson's correlation analysis showed a strong negative correlation between serum albumin level and SOFA score (correlation coefficient (r) = -0.78, p = 0.001). Linear regression analysis confirmed an inverse relationship between serum albumin levels and SOFA scores (beta coefficient = -2.70, p = 0.002).

Conclusions

In the present study, serum albumin level was noted as a reliable predictor of sepsis severity in ICU patients. Lower serum albumin levels were associated with higher SOFA scores, indicating more severe sepsis. This study supports the use of serum albumin as a simple and cost-effective biomarker for early identification of sepsis severity, particularly in resource-limited settings.

## Introduction

Sepsis is defined as a life-threatening organ dysfunction caused by a dysregulated host response to infection [1]. Sepsis is characterized by the presence of infection and of two or more Systemic Inflammatory Response Syndrome (SIRS) criteria: body temperature >38°C or <36°C, heart rate >90 beats per minute, respiratory rate >20 breaths per minute or PaCO2 <32 mmHg, and white blood cell count >12,000 cells/mm³ or <4,000 cells/mm³. Therefore, it’s possible that patients with sepsis can present with the following symptoms and signs including fever, tachycardia, tachypnea, altered mental status, reduced urine output, and hypotension based on its severity. However, its diagnosis can only be confirmed after specific investigations and documented infection [2,3].

Sepsis poses a significant burden on healthcare systems globally. All over the world, sepsis affects over 30 million people annually, resulting in approximately 6 million deaths [4]. The United States alone reports over 1.7 million sepsis cases annually, with a mortality rate of 15% [5]. In Pakistan, sepsis affects up to 45% of ICU admissions, with a mortality rate of 45.6% [6]. Compared to neighboring countries, according to the World Health Organization report (2019) on sepsis, Pakistan's sepsis burden is higher than India (34.6%) and Bangladesh (32.6%), but lower than Afghanistan (55.6%). Sepsis imposes significant economic burdens, with estimated annual costs exceeding 24 billion USD in the United States. Survivors often experience long-term cognitive, physical, and emotional impairments along with chronic diseases [5,7].

There are various known causes of sepsis worldwide and some major causes include pneumonia (primarily community-acquired pneumonia), intra-abdominal infections (mainly due to appendicitis, cholecystitis, and intestinal perforation), urinary tract infections (often caused by Escherichia coli), and skin and soft tissue infections (including cellulitis and necrotizing fasciitis) [4-6, 8].

Current treatment modalities for sepsis focus on early recognition and antibiotic administration, fluid resuscitation, vasopressor support, and organ support (e.g., mechanical ventilation) [9].

Diagnosis of sepsis is complex due to many reasons such as initial symptoms of sepsis can be nonspecific, lack of a definitive biomarker, overreliance on the SOFA (Sepsis-related Organ Failure Assessment) scoring system that can be cumbersome to calculate, and has limitations as well, difficulty in distinguishing sepsis from non-infectious systemic inflammatory response syndrome (SIRS), and limited sensitivity of blood cultures [1,10].

Even though different scoring systems such as sequential Organ Failure Assessment (SOFA) and Acute Physiology and Chronic Health Evaluation (APACHE) II, have been used in the assessment of severity and prognosis of sepsis. However, the influence of a great number of factors including age, comorbidities, immunosuppression, different infection sites and causative pathogens, antibiotics resistance, fluid inadequacy, and poor nutritional status, on sepsis prognosis and severity makes the use of these scoring systems complex and challenging [11-13]. Therefore, simple and cost-effective predictors of the severity and prognosis of sepsis are required to reduce the burden of sepsis all over the world.

Serum albumin level is emerging as a reliable predictor of prognosis and severity of sepsis especially in resource-limited countries [14-17]. Although the role of serum albumin level in the prediction of sepsis severity has been investigated in different regions of the world, in Pakistan, research work on the correlation between serum albumin level and severity of sepsis is limited. Therefore, this current study aims to evaluate the correlation between the serum albumin level and the severity of sepsis in the intensive care unit (ICU) admitted patients.

## Materials and methods

Study design and study population

We performed this cross-sectional study in the Intensive Care Unit (ICU) of Benazir Bhutto Hospital (BBH), Rawalpindi, Pakistan. A total of 201 patients were admitted to the ICU and diagnosed with sepsis during the period of one year from March 2022 to April 2023, were enrolled in the study through consecutive sampling techniques and devised inclusion and exclusion criteria. WHO sample size calculator was applied for the calculation of the sample size for the current study. A Turkish study with a prevalence of sepsis of 10.90% was used as a reference study and we maintained the power of the study at 80% whereas the confidence interval and margin of error were 95% and 5%, respectively [3].

Inclusion and exclusion criteria

All patients with either gender, age 18 years or above, measured serum albumin level, complete medical record, known etiology of infection, confirmed diagnosis of sepsis, and who were admitted to the intensive care units (Medical and surgical ICUs) within the last 24 hours, were included in the study. Whereas patients with age less than 18 years, suspected sepsis, do not resuscitate (DNR) orders (Pakistan Medical and Dental Council guidelines were used for DNR orders), pregnancy, history of treatment with the infusion of human albumin before the onset of sepsis, malnutrition, and known pre-existing comorbidities such as diabetes mellitus, hypertension, chronic kidney disease, chronic liver disease, congenital or heart disease, autoimmune disease, and malignancy and who were reluctant to enroll themselves in research, were excluded from the current study.

Ethics

For the current study ethical approval from the Ethics Review Board at Benazir Bhutto Hospital in Rawalpindi, Pakistan was obtained (approval number: BBH.ERB.283.229). Additionally, written informed consent was collected from each participant following a thorough explanation of the study's objectives and procedures, ensuring their understanding and voluntary involvement.

Primary outcome and secondary outcomes

The primary outcome of the current study was a correlation between serum albumin levels and the severity of sepsis using SOFA (Sequential Organ Failure Assessment) scores in ICU patients. At the same time, there were three secondary outcomes. The first was to identify the common causes of sepsis in ICU patients. Second was the comparison of serum albumin levels among three groups of study participants which included the sepsis group, severe sepsis group, and septic group. Third was the determination of the predictive capacity of serum albumin levels for sepsis severity progression including prediction of organ failure development.

Sepsis and severity of sepsis and serum albumin level

Systemic inflammatory response syndrome (SIRS) and sepsis were diagnosed according to the American College of Chest Physicians (ACCP) and the Society of Critical Care Medicine (SCCM). SIRS is a clinical condition characterized by a systemic inflammatory response to an insult, which can be infectious or non-infectious. SIRS is defined by the presence of two or more of the following criteria: 1) Body temperature either greater than 38°C (100.4°F) or less than 36°C (96.8°F); 2) Heart rate of more than 90 beats per minute; 3) Respiratory rate of more than 20 breaths per minute or PaCO2 less than 32 mmHg; 4) Complete blood count indicating any of these pictures such as white blood cell counts with greater than 12,000 cells/mm³, less than 4,000 cells/mm³ or more than 10% immature neutrophils bands. Sepsis was defined by the presence of at least two SIRS criteria and documented infection. These definitions for SIRS and sepsis have been used around the globe and in our study setting as well [2,4]. The severity of sepsis was assessed using the Sequential Organ Failure Assessment (SOFA) scoring system. The SOFA score is a widely used, validated tool to evaluate organ dysfunction in critically ill patients. The score ranges from 0 to 24, with higher scores indicating greater organ dysfunction (increased severity of sepsis). The SOFA score was calculated based on the dysfunction of six organ systems, each system dysfunction scored from 0 to 4 points, depending on the severity of the dysfunction. Assessment of the six organ systems including respiratory, cardiovascular, hepatic, coagulation, renal, and neurological was done using the specific parameters of these systems such as PaO2/FiO2 ratio (partial pressure of oxygen 2/fraction of inspiration oxygen 2), hypotension and vasopressor requirement, bilirubin level, platelet count, creatinine level and urine output, and Glasgow Coma Scale (GCS) score, respectively. Patients were categorized into three groups based on their total SOFA score. Patients were labeled to have sepsis, severe sepsis, and septic shock based on their SOFA score according to this order of SOFA scores such as 3-5 score (sepsis), 6-11 score (severe sepsis), and 12-24 score (septic shock) respectively. The SOFA score system has been used in different studies all over the world. However, we also calculated the Cronbach's alpha of the questionnaire for the severity of sepsis for 55 responses to assess its reliability in our population, and it was 0.80 which indicates great reliability in our setting [3, 12, 13, 18].

Sample collection for the required investigations

For the determination of blood level of the various parameters such as albumin (3.5-5.5 g/dL), total bilirubin (0.2-1.3 mg/dL), creatinine (0.7-1.3 mg/dL for men while 0.6-1.1 mg/dL for women), platelets count (150.0-450.0 × 10^9^/L), and PaO2/FiO2 ratio (400 or above mmHg via ABG) samples of all patients were taken by the registered nurses according to the protocols of the hospital that were made following the standards established by the College of American Pathologists (CAP).

Data collection

For this study, data collection was facilitated through a self-designed questionnaire. It had three distinct components. The first part was about the demographic details (age and gender), previous medical history (history of human albumin-related treatment and pre-existing comorbidities), and physical examination findings (temperature, heart rate, respiratory rate, blood pressure/mean arterial pressure (MAP), and GCS score mainly) for each patient. The second component was related to the reports of the required investigations. The third component was regarding the total score calculation of the Sequential Organ Failure Assessment (SOFA) score by using the information from the first two components.

Data analysis

Statistical analysis was performed using IBM SPSS Statistics, Version 25 (Released 2017; IBM Corp., Armonk, New York, United States). Quantitative data were summarized as mean ± standard deviation while qualitative data were expressed as frequencies and percentages. Comparisons among the three study groups were made using the One-way ANOVA test for numerical variables and the chi-squared test for nominal variables. The relationship between serum albumin levels and SOFA scores was examined using Pearson's correlation analysis. Additionally, a linear regression model assessed the predictive value of serum albumin levels for SOFA scores. Statistical significance was set at p < 0.05.

## Results

**Figure 1:**
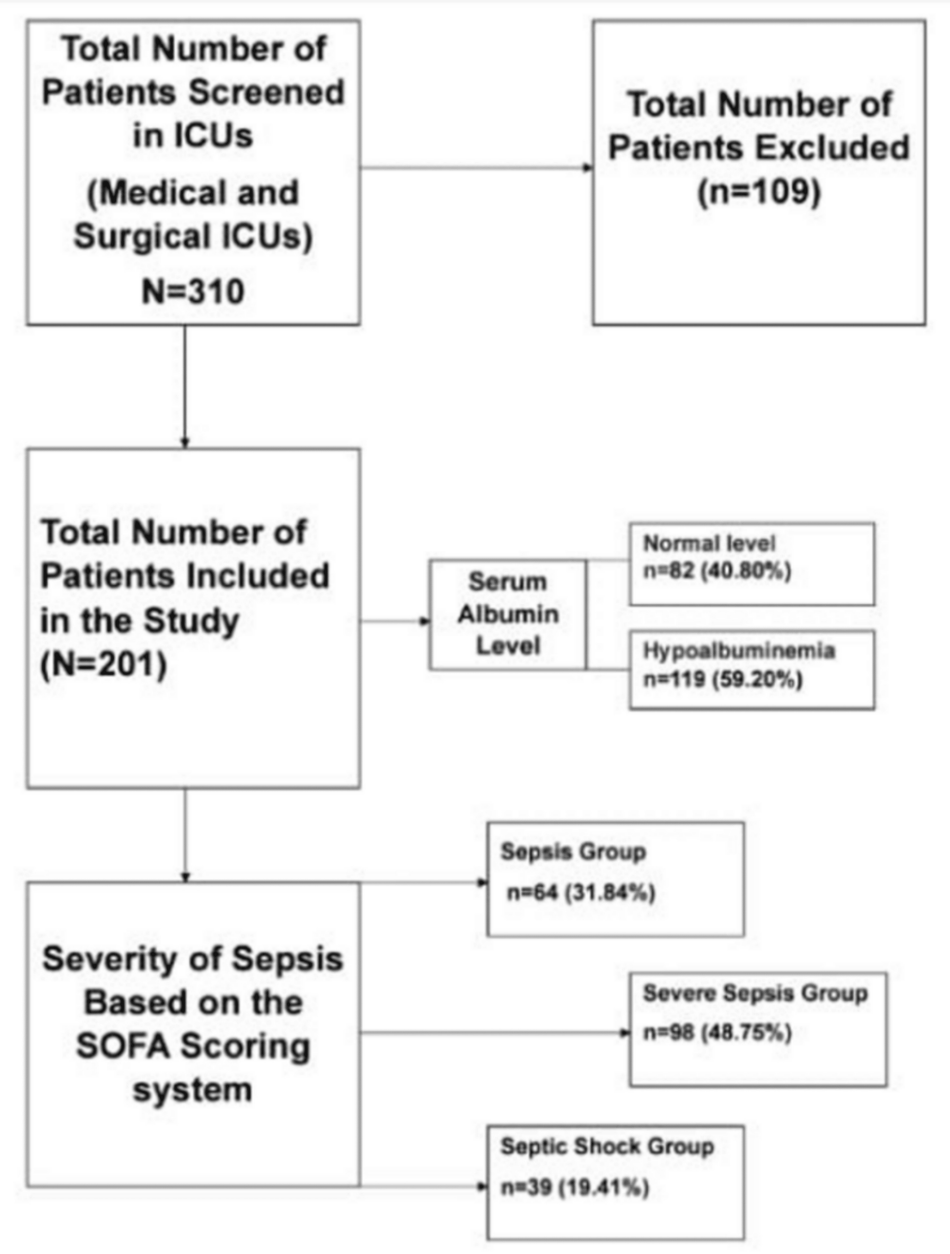
Flowchart of the Study

Of 201 patients, 64 (31.84%) patients had sepsis while 98 (48.75%) and 39 (19.41%) patients had severe sepsis and septic shock, respectively. Hypoalbuminemia was present among 119 (59.20%) patients whereas 82 (40.80%) patients had normal albumin levels. Most common cause of sepsis among the study population was respiratory tract infection (n=145, 72.18%), followed by urinary tract infection (n=32, 15.94%), intra-abdominal infections (n=18, 8.90%), skin infection (n=4, 1.99%), and bone infection (n=2, 0.99%) (Figure 1).

Table 1 shows the study population's characteristics including demographic and clinical. It has also revealed statistically significant differences among the three study groups in several key variables, including total bilirubin, serum creatinine, platelet count, PaO2/FiO2 ratio, mean arterial pressure, Glasgow Coma Scale score, SOFA score, serum albumin level, and the frequency of normal and low albumin levels (p < 0.05). In contrast, no significant differences were observed among the groups regarding age and gender (p > 0.05).

**Table 1:** Demographic and clinical features of the study population along with one-way ANOVA test and Chi-squared test analysis N: Study population size; n: Sample size for each group or category; %: Percentage; Mean ± SD: Mean value with standard deviation; p < 0.05: Statistically significant; F-value: A test statistic for ANOVA test; X²-value/chi-square value: A test statistic for Chi-Square test; SOFA: Sequential Organ Failure Assessment; PaO2: Partial Pressure of Arterial Oxygen; FiO2: Fraction of Inspired Oxygen. In the second last column, test statistic values with the sign of (+) are of the ANOVA test while test statistic values with the sign of (*) are of the Chi-square test. Similarly, p-values with the sign of (+) are of the ANOVA tests while p-values with the sign of (*) are of the Chi-square test. Furthermore, p-values with (S: Significant) are statistically significant whereas p-values with (IS: Insignificant) are statistically insignificant.

Variables, Patients with Sepsis (N=201)		Expression of Variables	Severity of Sepsis			One-way ANOVA test and Chi-Square test Analysis	
			Sepsis Group, n=64 (31.84%)	Severe Sepsis Group, n=98 (48.75%)	Septic Shock Group, n=39 (19.41%)	F-value and X^2^-value (Relevant Test Statistic)	p-values
Age (Years) Means ± SD		52.57±15.23	49.89±10.65	51.87±11.85	54.88±11.34	2.63^+^	0.07^+^ (IS)
Total bilirubin level (mg/dL) Means ± SD		2.87±1.50	1.22±0.5	3.81±1.69	4.70±2.49	13.19^+^	0.002^+^ (S)
Serum Creatinine level (mg/dL) Means ± SD		2.90±2.07	2.10±0.59	3.38±2.32	4.79±1.10	12.15^+^	0.002^+^ (S)
Platelets count (×10^9^/L) Means ± SD		180.87±24.87	145.66±30.11	114.88±20.45	102.57±14.82	15.67^+^	0.003^+^ (S)
PaO2/FiO2 ratio (mmHg) Means ± SD		388.93±50.76	320.87±44.98	299.07±45.87	240.40±44.76	23.91^+^	0.001^+^ (S)
Mean Arterial Pressure (mmHg) Means ± SD		90.56±8.56	86.99±6.22	78.34±8.09	70.65±12.90	14.29^+^	0.002^+^ (S)
Glasgow Coma Scale Score Means ± SD		11.23±3.50	12.45±2.12	10.67±3.56	7.24±5.46	10.39^+^	0.002^+^ (S)
SOFA Score Means ± SD		6.23±2.88	4.22±0.45	9.34±1.37	14.85±2.45	64.51^+^	0.002^+^ (S)
Serum Albumin level (g/dL) Means ± SD		3.22±1.40	3.40±1.69	2.95±2.44	1.90±0.78	8.62^+^	0.001^+^ (S)
Gender	Male n (%)	124 (61.69%)	40 (62.50%)	63 (64.28%)	21 (53.84%)	3.84^*^	0.05^*^ (IS)
	Female n (%)	77 (38.31%)	24 (37.50%)	35 (35.72%)	18 (46.16%)		
Serum Albumin Level Status	Normal level n (%)	82 (40.80%)	30 (46.87%)	41 (41.84%)	11 (28.20%)	7.14^*^	0.03^*^ (S)
	Hypoalbuminemia n (%)	119 (59.20%)	34 (53.13%)	57 (58.16%)	28 (71.80%)		

Table 2 indicates Pearson’s correlation analysis between the serum albumin levels and the severity of sepsis in the study population via correlation coefficient. The correlation coefficient was strongly negative and statistically significant. The negative correlation coefficient means that with the decrease in serum albumin level, the SOFA score goes up (sepsis severity increases).

**Table 2:** Correlation between serum albumin levels and severity of sepsis in the study population N: Study population sample size; n: Sample size for each group or category; %: Percentage; Mean ± SD: Mean value with standard deviation; p < 0.05: Statistically significant; (S): Statistically significant p-value.

Variables, N=201	Severity of Sepsis			One-way ANOVA test	Pearson’s Correlation	
	Sepsis Group	Severe Sepsis Group	Septic Shock Group	p-value	Correlation Coefficient (r)	p-value
SOFA Score Means ± SD	4.22±0.45	9.34±1.37	14.85±2.45	0.002	-0.78	0.001
Serum Albumin levels Means ± SD	3.40±1.69	2.95±2.44	1.90±0.78	0.001		

Table 3 shows that the simple linear regression model yielded statistically significant results, as evidenced by a highly significant F-test with a p-value less than 0.000. The model's goodness of fit was further confirmed by a high R-squared value of 0.82 (82%), indicating that the model explained a substantial proportion of the variance in the data. Notably, the beta coefficient for serum albumin levels was negative and statistically significant, indicating an inverse relationship between serum albumin levels and SOFA scores. Specifically, this means that higher serum albumin levels were associated with lower SOFA scores, corresponding to less severe sepsis. Conversely, lower serum albumin levels were linked to higher SOFA scores, indicating more severe sepsis.

**Table 3:** Findings of simple linear regression model for the study variables Mean ± SD: Mean value with standard deviation; CI: Confidence Interval; p < 0.05: Statistically significant; (S): Statistically significant. R2 value was 0.82 (82.00%). The Regression Model was significant as p-value of F test was ˂0.000 (Significant)

Variables	SOFA Score Mean ± SD	Serum Albumin level (g/dL) Mean ± SD	Unstandardized Regression Coefficient (β)	95% CI	p-value
	6.23±2.88	3.22±1.40	-2.70	-1.55 to -4.40	0.002

## Discussion

Sepsis, a fatal condition characterized by organ dysfunction due to a dysregulated host response to infection, poses a significant burden on healthcare systems globally. In this present study, we have acquired significant information regarding the correlation of serum albumin levels with the severity of sepsis in ICU patients. Moreover, this study has also examined the differences in the means of various study parameters such as total bilirubin, serum creatinine, platelet count, PaO2/FiO2 ratio, mean arterial pressure, Glasgow Coma Scale score, SOFA score, serum albumin level, and the frequency of normal and low albumin levels among the three study groups including patients with sepsis, severe sepsis, and septic shock.

In the present study population, 64 (31.84%) had sepsis, 98 (48.75%) had severe sepsis, and 39 (19.41%) had septic shock. Lower frequencies of sepsis (n=163, 10.87%), severe sepsis (n=260, 17.30%), and septic shock (n=203, 13.50%) have been noted in a Turkish study [3]. The incidence of hypoalbuminemia was 59.20% (n=119) among the study population. An Indian study has shown hypoalbuminemia in 56.90% (n=58) of its study population [15]. Differences in the severity of sepsis and hypoalbuminemia in the current study population from the studies from different countries could be due to many factors including healthcare infrastructure, infection control practices, microbial epidemiology, population demographics, comorbidities and underlying health conditions, and environment. The most common cause of sepsis among the study population was respiratory tract infection, followed by urinary tract infection, intra-abdominal infections, skin infection, and bone infection. Similar etiology of the sepsis has been presented in different studies [2-6].

Regarding the demographic characteristics of the study population, it was noted that with the increase in age, the severity of sepsis was also increased. Patients with sepsis (49.89±10.65 years) had lower mean age in contrast to patients with severe sepsis (51.87±11.85 years) and septic shock (54.88±11.34 years). Similarly, male gender (n=124, 61.69%) was dominant in the study participants. Different studies have also reported alike findings about the demographic characteristics of the patients with sepsis [12,14].

Regarding the differences in the means of main study variables, statistically significant differences were observed in the total bilirubin, serum creatinine, platelet count, PaO2/FiO2 ratio, mean arterial pressure, Glasgow Coma Scale score, SOFA score, and serum albumin level, across three study groups such as patients with sepsis, severe sepsis, and septic shock (p < 0.05). Furthermore, a significant difference in the frequency of normal and low albumin levels across three study groups was also noted (p < 0.05). In literature, various studies that had used SOFA score for the assessment of severity of sepsis, have supported these findings of the current study about the key parameters of the SOFA score system, SOFA score, and serum albumin levels [15-21].

Patients with increased severity of sepsis had lower serum albumin levels in contrast to those with less severe sepsis. This correlation was further backed and confirmed by the simple linear regression model. This finding suggests that serum albumin level is a significant predictor of sepsis severity (p=0.002). Many studies around the globe have endorsed this finding of the present study regarding the correlation between serum albumin level and the severity of sepsis. An Indian study has also presented alike results about the association between serum albumin level and sepsis severity. In this study, a significant association was observed between hypoalbuminemia and increased mortality in sepsis patients, with mortality rates of 29.3% and 11.4% in patients with and without hypoalbuminemia, respectively (p = 0.029) [15]. Likewise, a Turkish study has demonstrated a significant difference in the albumin levels between the survivors (albumin level=3.0 g/dL) and non-survivors (albumin level=2.6 g/dL) patients with sepsis (p=0.001) [16]. An American study has also revealed consistent findings about serum albumin levels and sepsis severity. It has shown that the probability of survival decreased by 63.4% when the serum albumin was ≤2.45 g/dl and by 76.4% when the serum albumin was ≤1.45 g/dl [17]. A Chinese study has also found that patients with lower serum albumin levels had more severe sepsis in comparison to patients with normal albumin levels. In that study, the patients’ group with the lowest albumin level (17.40±47 g/dL) had the highest score (12.0) on the SOFA scoring system [18]. Some studies from other parts of the world have also observed the importance of serum albumin levels in the determination of the severity of sepsis similar to the current study [19, 22]. The findings of the current study are consistent with previous research, supporting serum albumin level as a viable and trustworthy biomarker for evaluating the severity of sepsis.

Lower serum albumin levels significantly contribute to the increased severity of sepsis through several interconnected mechanisms. Firstly, albumin maintains oncotic pressure, preventing fluid shift from blood vessels to interstitial spaces. When albumin levels are low, oncotic pressure decreases, leading to increased vascular permeability, fluid loss from blood vessels, and subsequent edema formation [17]. Albumin plays a crucial role in blood coagulation by binding to and transporting coagulation factors, maintaining hemostasis. Impaired albumin levels disrupt coagulation factor transport, increasing the risk of bleeding and disseminated intravascular coagulation (DIC). Furthermore, albumin's anti-inflammatory properties are compromised when levels are low, allowing pro-inflammatory cytokines to exacerbate systemic inflammation. Lastly, albumin's antioxidant activity is impaired, enabling reactive oxygen species (ROS) to mediate tissue damage and enhance oxidative stress. Collectively, these mechanisms underscore the critical role of serum albumin in maintaining vascular integrity, coagulation homeostasis, and inflammatory balance, highlighting the importance of monitoring and supplementation of albumin in sepsis management [18, 19].

The findings of the current study have significant implications for clinical practice. Serum albumin level is a readily available, inexpensive, and easily interpretable biomarker that can facilitate early identification of sepsis severity. Regular monitoring of serum albumin levels may enable timely interventions, improving patient outcomes. Therefore, this study recommends the development of guidelines incorporating serum albumin level as a prognostic indicator for sepsis severity.

While this study contributes valuable insights, it has certain limitations. Notably, the cross-sectional design and single-center setting may restrict the broader applicability of our findings. Therefore, further research is warranted to validate these results and delve deeper into the underlying mechanisms driving the relationship between serum albumin levels and sepsis severity. Additional studies will help enhance the generalizability and depth of understanding in this area.

## Conclusions

In the present study, serum albumin level was negatively and significantly correlated with the severity of sepsis via the SOFA scoring system. Lower serum albumin levels were associated with increased SOFA scores which means increased severity of sepsis. The simple linear regression model also confirmed an inverse relationship between serum albumin levels and SOFA scores. These findings suggest that serum albumin level could be used as an efficient and cost-effective predictor of sepsis severity in ICU patients. Our study also recommends the regular monitoring of serum albumin levels among ICU patients for timely interventions and better patient outcomes. Clinicians should consider serum albumin levels in conjunction with other diagnostic tools to assess sepsis severity.
